# Development of Ternary Ti-Ag-Cu Alloys with Excellent Mechanical Properties and Antibiofilm Activity

**DOI:** 10.3390/ma15249011

**Published:** 2022-12-16

**Authors:** Genichi Togawa, Masatoshi Takahashi, Hiroyuki Tada, Yukyo Takada

**Affiliations:** 1 Division of Dental Biomaterials, Tohoku University Graduate School of Dentistry, 4-1 Seiryo-machi, Aoba-ku, Sendai 980-8575, Japan; 2 Division of Oral Immunology, Tohoku University Graduate School of Dentistry, 4-1 Seiryo-machi, Aoba-ku, Sendai 980-8575, Japan

**Keywords:** mechanical property, biofilm, titanium alloy, dental implant, antibacterial activity

## Abstract

Titanium-20 mass% Silver (Ti-20%Ag) alloy can suppress biofilm formation on the surface. Unlike bactericidal agents, it does not kill bacteria; therefore, the healthy oral microflora remains undisturbed. To utilize the unique functions of this alloy and enable its use in the fabrication of dental prostheses that require relatively high strength, we added copper (Cu) as an alloying element to improve strength. This study aimed to develop ternary Ti-Ag-Cu alloys with excellent mechanical properties and antibiofilm activity. As a result of investigating the mechanical properties of several experimental alloys, the tensile strength, yield strength, and hardness of Ti-20%Ag-1%Cu and Ti-20%Ag-2%Cu alloys were improved by the solid-solution strengthening or hardening of the αTi phase. In addition, these alloys had the same ability to suppress biofilm formation as the Ti-20Ag alloy. Thus, Ti-20%Ag-1–2%Cu alloys can be used for fabrication of narrow-diameter dental implants and prostheses subjected to extremely high force, and these prostheses are useful in preventing post-treatment oral diseases.

## 1. Introduction

Titanium (Ti) is a metallic material with excellent biocompatibility and good corrosion resistance and is used as an implant material in dentistry. Currently, Ti is rarely used in superstructures. However, to reduce the potential difference and avoid galvanic corrosion, it is desirable that the superstructure and abutment are fabricated using Ti-based alloys. In recent years, the demand for narrow-diameter and short implants has increased; thus, miniaturization of implant bodies is required. However, the strength of Ti and the currently used Ti alloys is insufficient, and problems, such as breakage of the implant body due to inadequate strength, are often encountered [1,2]. Thus, they cannot be used for fabrication of dental prostheses that require a relatively high strength, such as long-span bridges [3,4,5]. Therefore, the development of Ti alloys with higher strength is required.

Due to the excellent biocompatibility of Ti, it is easy for bacteria to breed and form biofilms on its surface [6,7]. Biofilm formation on dental implants and prostheses causes various oral diseases, such as peri-implantitis, secondary caries, and periodontitis. Ti is also used for the framework of dentures, and there are some clinical reports that denture plaque adheres more easily to Ti than to other metals [8,9]. Therefore, many studies have been conducted to impart antibacterial properties to Ti surfaces through surface modification, such as drug loading [10], anodization and ion implantation [11], and antimicrobial peptide fixation [12].

Developmental research conducted on various Ti alloys [13,14,15,16,17] has revealed that a Ti–silver (Ag) alloy containing 20 mass% Ag (hereafter, “%” represents “mass%”) has a unique property of suppressing biofilm formation on its surface [13]. In contrast, the Ti-20%Ag alloy did not show bactericidal activity in the antibacterial activity test [13]; while the Ti-20%Ag alloy had good corrosion resistance, it hardly released Ag ions [18,19,20]. An immersion test conducted in a simulated body fluid revealed that, similarly to Ti, Ti-20%Ag possessed the ability to form calcium phosphate spontaneously and was evaluated to have good biocompatibility [21]. Thus, the Ti-20%Ag alloy has antibacterial properties that are extremely safe for living organisms and suppresses biofilm formation without bactericidal activity. There are a large number and many types of microorganisms in the oral cavity; these bacteria constitute the oral flora and are important for protecting the oral environment from the invasion of pathogenic bacteria [22,23]. Antimicrobial agents, such as antibiotics and antimicrobial materials, can kill or weaken the beneficial indigenous bacteria, and their long-term use can cause microbial substitution. Therefore, the ability of Ti-Ag alloys to suppress biofilm formation is a valuable function for dental materials.

The strength of the Ti-20%Ag alloy is more than 1.6 times higher than that of Ti [15], and its yield strength and elongation satisfied the criteria for type 4 metallic materials according to the ISO classification [24], indicating that Ti-20%Ag can be used in the fabrication of various prostheses, such as superstructures and bridges. However, the strength of the Ti-20%Ag alloy was slightly lower than that of hardened type 4 gold alloys [25], which are also classified as ISO type 4, and that of cobalt–chromium alloys [26], which are classified as ISO type 5. A further increase in the strength of Ti-20%Ag is required to enable their use in the fabrication of large prostheses that require higher strength, such as long-span bridges and thin metal denture bases.

In this study, the Ti-20%Ag alloy was converted into a ternary alloy to improve its strength while taking advantage of its unique ability to suppress biofilm formation. Copper (Cu), a well-known metallic element that constitutes dental alloys with Ag, was selected as the alloying element. Ag and Cu are classified as β-stabilizing and β-eutectoid elements [27]. Although the mechanism of the ability of Ti-Ag alloys to suppress biofilm formation has not yet been clarified, Cu has antibacterial properties similar to those of Ag [28,29,30]. If the same effect can be obtained by adding Cu to Ti, it may lead to enhancement rather than maintenance of its ability to suppress biofilm formation. Studies on binary Ti alloys have shown that Cu can improve the strength of Ti with fewer additions than Ag [15]. The tensile strength of the Ti-5%Cu alloy was approximately 700 MPa, which was approximately twice that of Ti [15]. However, the addition of Cu greatly reduced the elongation of Ti, and the elongation of the Ti-10%Cu alloy was less than 1% [15]. Therefore, the amount of Cu added to the alloys in this study was less than 10%. Since the elongation of Ti-20%Ag is as high as 19% [15], it is possible to increase the strength while maintaining the required elongation as a dental alloy through the addition of a small amount of Cu.

Thus, ternary Ti-Ag-Cu alloys with 20% Ag and 1–10% Cu were designed in this study, and the microstructure, mechanical properties, antibiofilm activity, and antibacterial activity of these alloys were investigated to develop dental Ti alloys with excellent mechanical properties and antibiofilm activity.

## 2. Materials and Methods

### 2.1. Preparation of Alloy Specimen

The compositions of the experimental Ti-Ag-Cu alloys used in this study were as follows: Ti-20%Ag-1%Cu (20Ag1Cu), Ti-20%Ag-2%Cu (20Ag2Cu), Ti-20%Ag-5%Cu (20Ag5Cu), and Ti-20%Ag-10%Cu (20Ag10Cu). Ti (>99.8%, grade S-90, Osaka Titanium Technologies, Amagasaki, Japan), Ag (>99.9%, Hirano Seizaemon Shoten, Tokyo, Japan), and Cu (>99.99%, Research Institute for Electric and Magnetic Materials, Sendai, Japan) were weighed according to their designed compositions and melted in an argon arc melting furnace to produce 30 g alloy ingots. Similarly, a Ti ingot was also prepared as the control.

Plate-shaped and dumbbell-shaped samples were prepared by casting each ingot) into a mold made of magnesia investment (Symbion-TC, i-Cast Co., Ltd., Kyoto, Japan) using a Ti casting machine (Autocast HC-III, GC, Tokyo, Japan. Each surface of the plate-shaped casting was ground to a depth of 300 μm using waterproof silicon carbide abrasive papers to remove the surface-hardened layer. Each surface was polished to 800 grit, and the polished specimens (10 mm × 15 mm × 3 mm) were used for various tests. The dumbbell-shaped specimens, which were 3 mm in diameter and 15 mm in gauge length, were used for the tensile test without removing the hardened layer on the surface.

### 2.2. X-ray Diffraction Analysis

X-ray diffraction (XRD) was performed with Cu Kα radiation (30 kV, 10 mA) using an X-ray diffractometer (D2 PHASER; Bruker, Tokyo, Japan). The measurement conditions were 2*θ* = 30°–80° with a scanning step width of 0.03°. The Crystallography Open Database was used for phase identification.

### 2.3. Microstructural Observation

The plate-shaped specimens were mirror-polished with a diamond suspension and etched with a hydrofluoric–nitric acid solution. Subsequently, their metallographic structures were observed using an optical microscope (ECLIPSE MA-200, Nikon, Tokyo, Japan).

### 2.4. Hardness Test

The plate-shaped specimens were mirror-polished using a diamond suspension. Subsequently, the Vickers hardness of the specimens was evaluated using a microhardness tester (HM-221, Mitutoyo, Kawasaki, Japan) with a load of 1.961 N and a dwell time of 15 s (*n* = 10).

### 2.5. Tensile Test

Tensile tests followed the test procedure specified by ISO 22674 [24]. The tests were performed using a universal testing machine (AG-IS, Shimadzu, Kyoto, Japan) with a crosshead speed of 0.5 mm/min (*n* = 6). The ultimate tensile strength, yield strength (proof strength of 0.2% nonproportional extension), and elongation after fracture were determined. A scanning electron microscope (SEM; JSM-6060, JEOL, Tokyo, Japan) was used to observe the fractured surfaces.

### 2.6. Surface Roughness Test

The surface roughness of the specimens polished to 800 grit was measured using a surface profilometer (Surfcom 480A, Tokyo Seimitsu, Tokyo, Japan) (*n* = 5). The evaluation length was 4.0 mm, the stylus speed was 0.6 mm/s, and the cutoff value was 0.8 mm. The surface roughness was characterized by the height parameter *Ra*.

### 2.7. Biofilm Formation Test

Two strains of mutans streptococci, *Streptococcus mutans* (ATCC25175) and *Streptococcus sobrinus* (ATCC27351), were used in the biofilm formation test. These bacteria were inoculated in the brain-heart infusion (BHI) broth and pre-cultured overnight, at 37 °C. The bacteria in the BHI broth were transferred to the BHI liquid medium and anaerobically cultured, at 37 °C, until the early log growth phase (optical density (OD) = 0.15 at 600 nm).

**Figure 1:**
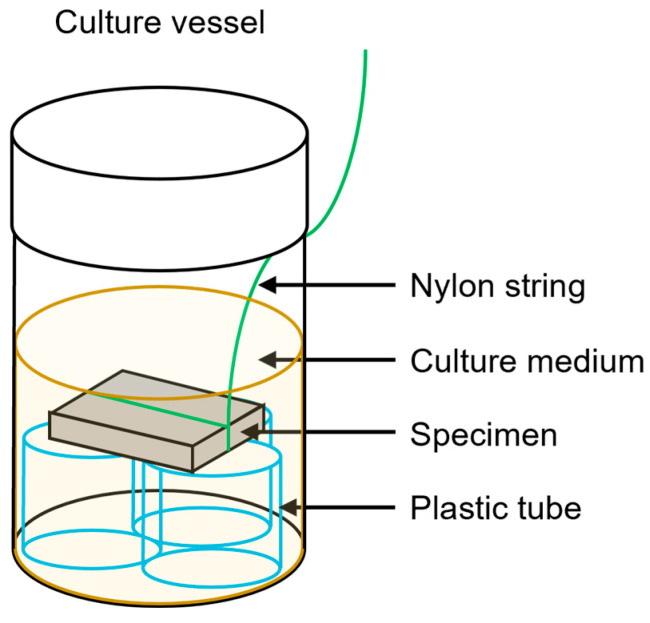
Schematic drawing of the experimental apparatus.

After polishing the specimen to 800 grit, it was ultrasonically cleaned using 99.5% ethanol for 2 min. A nylon string was wound around each specimen. Each culture vessel was filled with 15 mL of t-tryptone soya liquid medium containing 5% sucrose and 20 μL of the bacterial culture. An autoclaved specimen was immersed in the culture vessel and placed horizontally, separated from the bottom of the vessel (Figure 1). The culture vessel was maintained under anaerobic conditions, at 37 °C. After 15 h, the specimens with the adhered bacterial accumulation were removed from the culture vessel. The bacterial accumulation was removed via gentle vibration in pure water, and the bacteria firmly adhering to the specimens were scraped with a sterile plastic spatula. These samples were suspended in pure water, and the amount of bacteria was estimated by measuring the OD at 600 nm with a spectrophotometer (*n* = 12). The amount of bacteria was defined as the amount of biofilm formed.

### 2.8. Antibacterial Activity Test

Two strains of mutans streptococci, the same as that in the biofilm formation test, were used for the antibacterial activity test. These bacteria were inoculated in the BHI broth and pre-cultured overnight, at 37 °C. The bacteria in the BHI broth were transferred to the BHI liquid medium and anaerobically cultured, at 37 °C, until the early log growth phase. The bacterial culture was washed three times by centrifuging (8500× *g* for 5 min at 4 °C) in phosphate-buffered saline (PBS). The collected bacteria were suspended in PBS to adjust the OD = 0.15 to 600 nm, and this bacterial suspension was subsequently used for the test.

We pipetted 5 μL of the bacterial suspension onto the surface of each autoclaved specimen. The bacterial suspension was covered with a 5 mm square sterile plastic film to spread the bacterial suspension on the surface. Each specimen was incubated at 37 °C and 90% relative humidity under aerobic conditions. After 2 h, the bacteria on the specimen were washed off and homogenized with 2 mL PBS in a sterile plastic bag, and 1 mL of the bacterial suspension was taken from the bag. The samples were serially diluted with PBS and spread onto the BHI agar plates. The plates were incubated at 37 °C for 2 days, and the number of colony-forming units (CFU) was counted (*n* = 16). The antibacterial activity (A) was calculated using the viable count after direct contact with Ti (B) and the viable count after direct contact with a Ti-Ag-Cu alloy (C) using Equation (1), which has been specified in the Japanese Industrial Standards (JIS) Z 2801 [31].

A = log_10_(B/C) (1)

An antibacterial effect was considered to be present if the antibacterial activity value was 2.0 or more.

### 2.9. Statistical Analysis

The obtained data were statistically analyzed with SPSS software version 28 using one-way ANOVA and Tukey’s HSD test at a significance level of α = 0.05.

## 3. Results

### 3.1. XRD and Metallography

**Figure 2:**
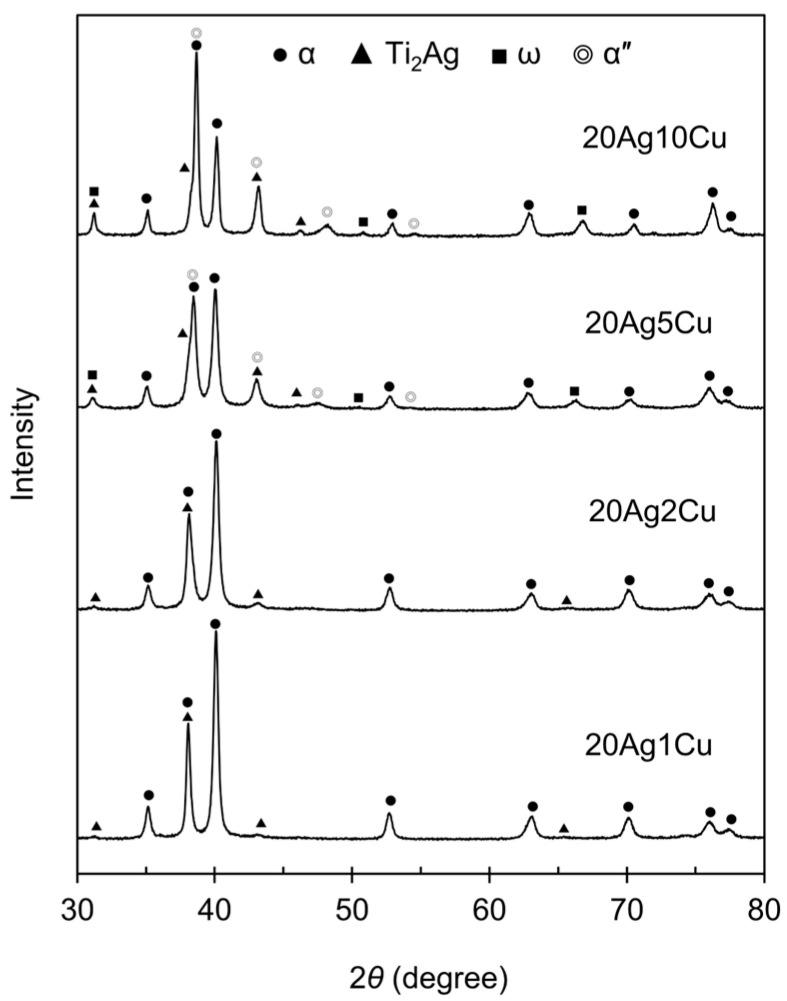
X-ray diffraction patterns of the Ti-Ag-Cu alloys.

The XRD patterns of the Ti-Ag-Cu alloys are shown in Figure 2. The XRD patterns of 20Ag1Cu and 20Ag2Cu showed peaks corresponding to αTi and Ti_2_Ag. The alloy phase was αTi + Ti_2_Ag. In the XRD patterns of 20Ag5Cu and 20Ag10Cu, peaks corresponding to ω and α″ were also observed in addition to αTi and Ti_2_Ag. No peaks corresponding to Ti_2_Cu were observed for any composition.

**Figure 3:**
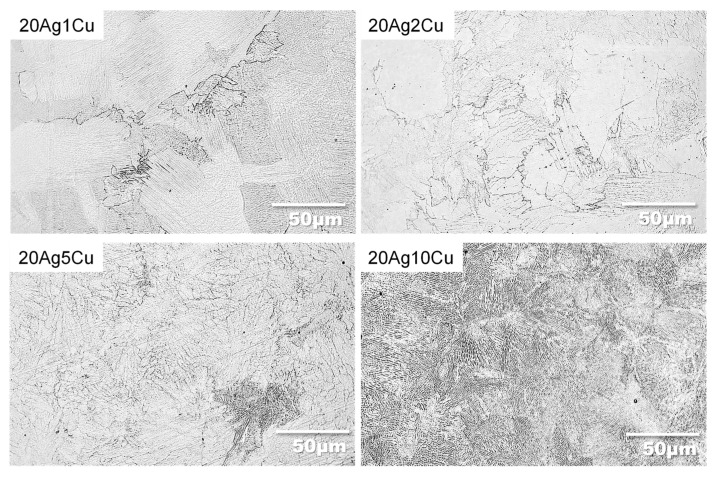
Microstructures of the etched Ti-Ag-Cu alloys.

Figure 3 shows the metallographic structure of the etched Ti-Ag-Cu alloys. In 20Ag1Cu and 20Ag2Cu, the acicular α was distributed homogeneously throughout the field of view. A few structures indicating Ti_2_Ag were observed. 20Ag5Cu had a fine acicular structure, whereas 20Ag10Cu had it all over the surface. A small amount of Ti_2_Ag was observed at the grain boundaries.

### 3.2. Hardness

**Figure 4:**
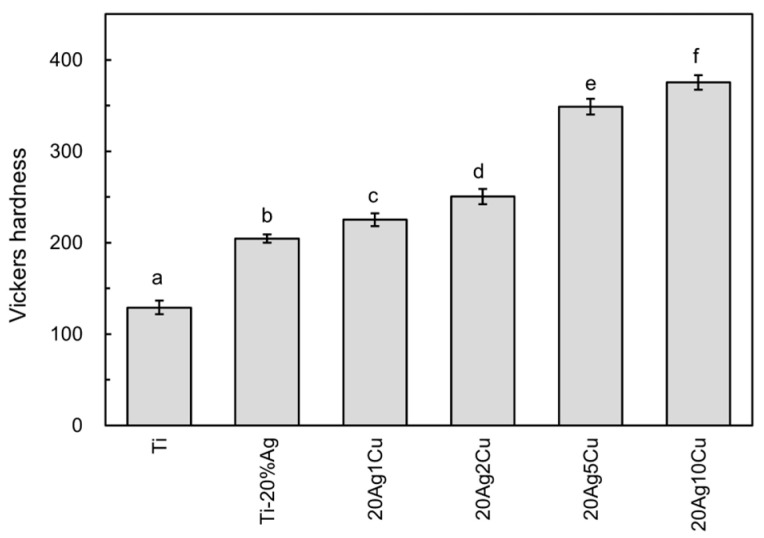
The Vickers hardness of the Ti-Ag-Cu alloys. Identical letters indicate a statistically insignificant difference (*p* > 0.05).

The Vickers hardness of the Ti-Ag-Cu alloys is shown in Figure 4, including the data for the Ti-20%Ag alloy obtained in a previous study [15]. The hardness of the Ti-Ag-Cu alloys were significantly higher than those of Ti and Ti-20%Ag (*p* < 0.01). The hardness of the Ti-Ag-Cu alloys increased as the Cu content increased, and significant differences were observed among all the compositions (*p* < 0.01). Compared with Ti-20%Ag, the hardness of 20Ag1Cu and that of 20Ag2Cu increased by approximately 10% and 20%, respectively. The hardness of the Ti-Ag-Cu alloy increased drastically when the amount of Cu was greater than 5%.

### 3.3. Tensile Test

**Figure 5:**
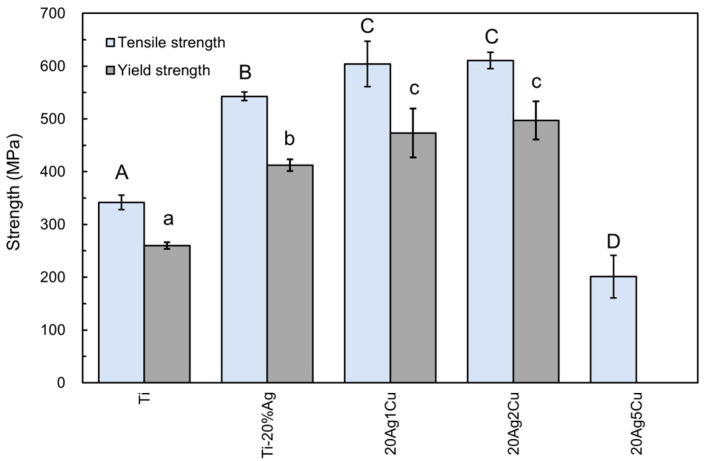
Tensile and yield strengths of the Ti-Ag-Cu alloys. Identical letters indicate a statistically insignificant difference (*p* > 0.05).

The tensile and yield strengths of the Ti-Ag-Cu alloys are shown in Figure 5, including the data for the Ti-20%Ag alloy obtained in a previous study [15]. The tensile and yield strengths of 20Ag1Cu and 20Ag2Cu were significantly higher than those of Ti-20%Ag (*p* < 0.05). The yield strength of 20Ag1Cu was greater than 450 MPa, and that of 20Ag2Cu was close to 500 MPa. The tensile strength of both alloys was approximately 600 MPa. However, the tensile strength of 20Ag5Cu was approximately 200 MPa, which is lower than that of Ti. The elongation of 20Ag5Cu was too small to determine its yield strength. Tensile testing of 20Ag10Cu could not be performed as 20Ag10Cu was brittle and broke during testing.

**Figure 6:**
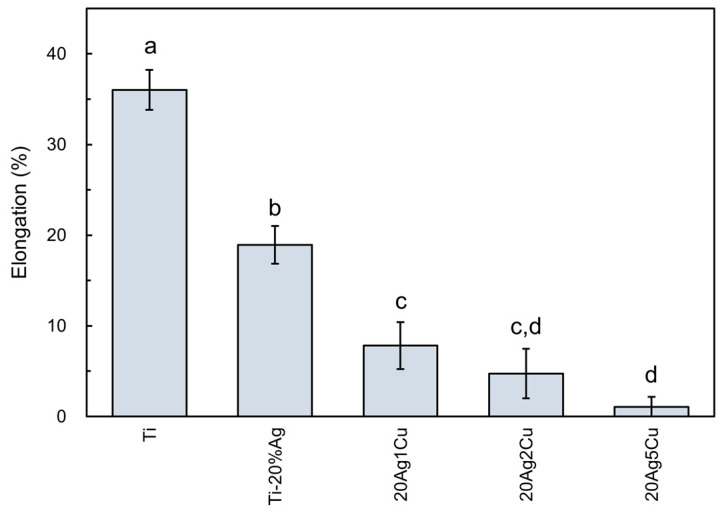
Elongation of Ti-Ag-Cu alloys. Identical letters indicate a statistically insignificant difference (*p* > 0.05).

The elongation of the Ti-Ag-Cu alloys are shown in Figure 6, including the data for the Ti-20%Ag alloy obtained in a previous study [15]. The elongation of the Ti-Ag-Cu alloy decreased as the Cu content increased: 8% for 20Ag1Cu and 5% for 20Ag2Cu. The elongation of 20Ag5Cu was less than 1%.

**Figure 7:**
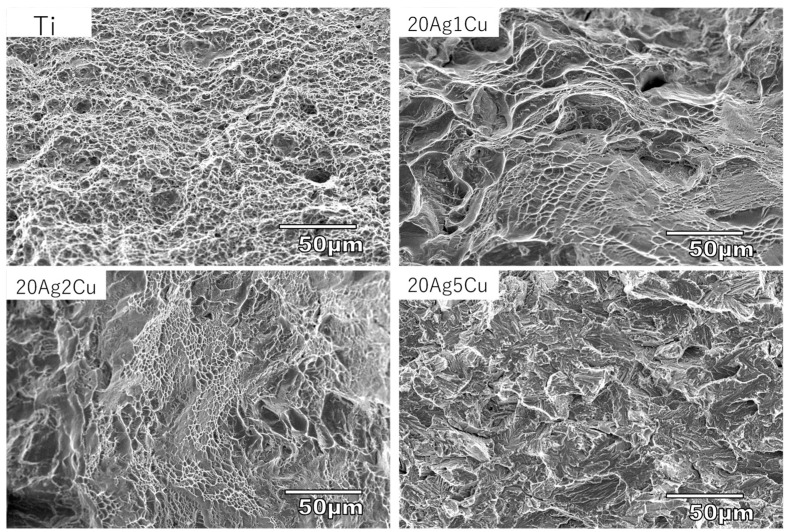
Scanning electron micrographs of the fracture surface of Ti-Ag-Cu alloys after tensile test.

Figure 7 shows the fracture surface of the alloys after tensile testing. Dimples indicating ductile fractures were observed on the entire surface of Ti. Dimples were partially observed on the fracture surfaces of 20Ag1Cu and 20Ag2Cu. The fracture surface of 20Ag5Cu exhibited cleavage without dimples.

### 3.4. Surface Roughness

**Figure 8:**
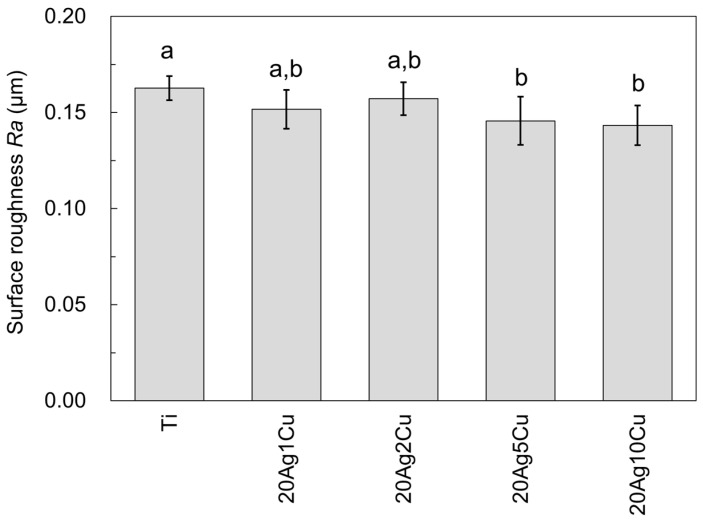
Surface roughness (*Ra*) of the Ti-Ag-Cu alloys. Identical letters indicate a statistically insignificant difference (*p* > 0.05).

Figure 8 shows the surface roughness (*Ra*) of the Ti-Ag-Cu alloys. The surface roughness of all the samples was less than 0.2 μm, with the surface roughness of Ti being the highest. The surface roughness of the Ti-Ag-Cu alloy decreased as the content of Cu increased. The surface roughness values of 20Ag5Cu and 20Ag10Cu were significantly lower than that of Ti (*p* < 0.05).

### 3.5. Biofilm Formation on Alloy Specimen

**Figure 9:**
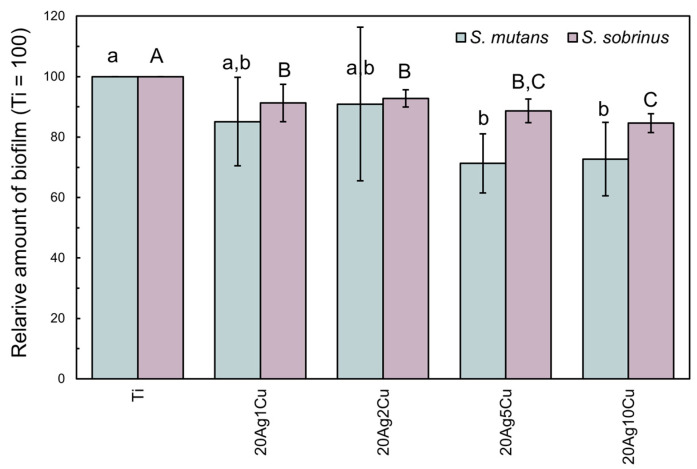
The relative amount of biofilm scraped from the surfaces of each specimen. Identical letters indicate a statistically insignificant difference (*p* > 0.05).

Figure 9 shows the biofilms formed by *S. mutans* and *S. sobrinus* firmly adhered to the surface of Ti and the Ti-Ag-Cu alloys. The amount of biofilm formed on the Ti-Ag-Cu alloys tended to decrease as the content of Cu increased. For *S. mutans*, the amount of biofilm formed on 20Ag5Cu and 20Ag10Cu was significantly lower than that formed on Ti (*p* < 0.01). For *S. sobrinus*, the amount of biofilm formed on all Ti-Ag-Cu alloys was significantly lower than that formed on Ti (*p* < 0.01).

### 3.6. Antibacterial Activity of the Alloy Specimen

**Figure 10:**
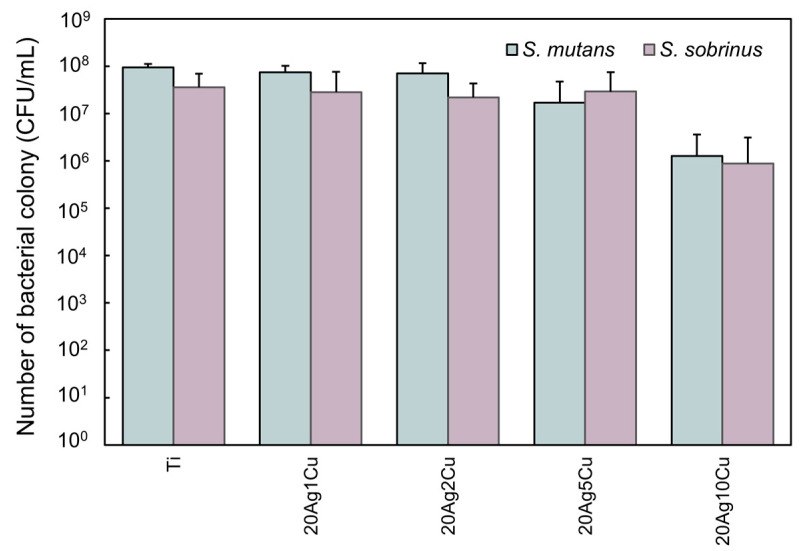
Viable count after direct contact with the metals.

Figure 10 shows the viable count after direct contact with the metals. For both bacteria, the viable count on the Ti-Ag-Cu alloy with up to 2% Cu content was the same as that on Ti. For *S. mutans*, the viable count on 20Ag5Cu was slightly decreased compared with that on Ti, whereas that on 20Ag10Cu was greatly decreased. In contrast, for *S. sobrinus*, the viable cell count on 20Ag5Cu was the same as that on Ti, whereas that on 20Ag10Cu was greatly decreased. All compositions exhibited antibacterial activity values of less than 2.0 and were judged to have no bactericidal activity.

## 4. Discussion

### 4.1. Alloy Phases

According to the equilibrium phase diagram of the binary system, the solid solubility limits of Ag and Cu in α-Ti are 7% at 600 °C and less than 1% at 500 °C, respectively [27]. In the Ti-Ag and Ti-Cu systems, the eutectoid reaction of the β→α+intermetallic compound (Ti_2_Ag/Ti_2_Cu) occurs at 855 °C and 790 °C, respectively [27]. Therefore, the addition of alloying elements exceeding the solid solubility limit precipitates the intermetallic compounds. Thus, the alloy phase of all Ti-Ag-Cu alloys designed in this study are expected to become αTi + Ti_2_Ag + Ti_2_Cu [32]. However, the XRD results varied, and no Ti_2_Cu peak was observed in any of the samples. The specimens used in this study were non-equilibrium solidification structures produced by dental casting and did not necessarily match the structures expected according to the equilibrium diagram. For example, β-stabilizing elements, such as Ag, Cu, Fe, and Mn, extend the α-β transformation temperature of titanium to the low-temperature side, making it easier to retain the β-phase at room temperature. By alloying Ti with more than 8% of Fe or more than 10% of Mn, the β phase can be retained at room temperature by dental casting [33,34]. However, unlike Fe and Mn, Ag and Cu are not strongly β-stabilizing; therefore, the β phase may not be retained upon quenching for any amount of Ag and Cu [35]. Some Ti alloys, such as Ti-Ag and Ti-Cu, undergo massive transformation to form a supersaturated solid solution that exceeds the solid solubility limit. Under cooling conditions for dental casting, up to 20% of Ag and up to 10% of Cu is solid-solved in α-Ti, and intermetallic compounds are not precipitated in binary Ti alloys [15,18]. Thus, the Cu added in this study would have been solid-solved in α-Ti. The XRD patterns of Ti-Ag-Cu alloys with more than 5% Cu showed ω (hexagonal) and α″ (orthorhombic) peaks, indicating metastable phases that may form during the β→α transformation process when cooled at a relatively fast rate. The addition of Ag and Cu expanded the β region to the low-temperature side; although the β phase was not retained, the metastable phase was retained. Corresponding to the change in the alloy phase, the microstructure also changed with the addition of ≥5% Cu, and a fine acicular structure was observed. Most of the Ti_2_Ag existed in grain boundaries. Even in the Ti-20%Ag alloy casting, Ti_2_Ag precipitated more at the grain boundaries and was rarely observed inside the grains [18].

### 4.2. Mechanical Properties

Compared with Ti-20%Ag, the strength and hardness of Ti-Ag-Cu alloys with 1–2% Cu were significantly improved. The yield strength of these alloys is approximately twice that of Ti. The alloy phases of both Ti-20%Ag and Ti-Ag-Cu alloys were α + Ti_2_Ag; however, the matrix of Ti-20%Ag was a binary solid solution, whereas the matrix of the Ti-Ag-Cu alloy was a ternary solid solution. In addition, the amount of solid-solution elements was larger in the Ti-Ag-Cu alloy than that in the Ti-20%Ag alloy. The solid-solution strengthening/hardening of αTi contributes greatly to the improvement in strength and hardness. Although the elongation of these Ti-Ag-Cu alloys was lower than that of Ti-20%Ag, many dimples were observed on the fracture surfaces, thereby proving their ductility.

Ti-Ag-Cu alloys with more than 5% Cu showed a significant increase in hardness, a decrease in strength, and a significant decrease in elongation. The changes in these mechanical properties may be attributed to the ω phase. When the ω phase occurs in titanium alloys, the material becomes hard and brittle; this is harmful in practice and is known as omega brittleness [36,37]. A cleavage fracture surface, which is characteristic of brittle fracture, was observed on the fracture surface of 20Ag5Cu, while 20Ag10Cu was too brittle for tensile testing. Thus, 20Ag5Cu and 20Ag10Cu were unsuitable for dental applications.

### 4.3. Antibiofilm Activity

The present study showed that while the Ti-Ag-Cu alloys inhibited biofilm formation on the surface, they were not bactericidal. These findings suggest that, similarly to the Ti-20%Ag alloy, the Ti-Ag-Cu alloys have antibiofilm activity that inhibits bacterial adhesion and colonization of the surface but does not affect bacterial viability.

Surface roughness is known to affect the adherence of bacteria. The adhesion of plaque, which is a typical example of biofilm in the oral cavity, to the material surface increases when the surface roughness exceeds 0.2 μm [38,39]. All samples in this study had a surface roughness of <0.2 μm and were clinically polished. Surface roughness is negatively correlated with hardness, and an increase in the hardness of the Ti-Ag-Cu alloys led to a decrease in surface roughness. It was observed that 20Ag5Cu and 20Ag10Cu had a significantly lower surface roughness than Ti, and the amount of biofilm formed was significantly lower than that of Ti. This suggests that the lower surface roughness may partly contribute to the reduced bacterial adhesion of the Ti-Ag-Cu alloy. However, for *S. sobrinus*, although the surface roughness of 20Ag1Cu and 20Ag2Cu did not differ significantly from that of Ti, the amount of biofilm formed on 20Ag1Cu and 20Ag2Cu was significantly lower than that formed on Ti. In addition, a previous study [13] showed that pure Ag and Ag-palladium (Pd) alloys, which had significantly lower surface roughness than Ti, did not exhibit any antibiofilm activity. These findings indicate that low surface roughness alone cannot explain the low bacterial adhesion of the Ti-Ag-Cu alloys.

In the antibacterial activity test, the Ti-Ag-Cu alloy was determined to have no antibacterial activity; however, the viable counts on 20Ag5Cu and 20Ag10Cu decreased compared with that on Ti. A decrease in the number of viable bacteria on the alloy surface may have led to a decrease in the amount of biofilm formed. However, in previous studies [13], pure Ag and Ag-Pd alloys showed strong antibacterial activity but did not reduce the amount of biofilm formed. A reduction in the number of viable bacteria is also considered a factor in the ability to suppress biofilm formation; however, this factor alone cannot explain it. Ti-30%Ag, which contains more Ag, did not decrease the number of viable bacteria [13]. The addition of Cu as an alloying element likely had a strong effect on the decrease in the number of viable bacteria. In the elution test for Ti-Cu alloys, a small amount of Cu ions were eluted from αTi depending on the Cu content, although the amount from αTi was very small compared with that from Ti_2_Cu [40]. A small amount of Cu ions eluted from the α-Ti of 20Ag5Cu and 20Ag10Cu possibly decreased the number of viable bacteria.

The micro-area potential differences on the surface of the alloys affect their antibacterial activity [41]. Fine Ti_2_Ag was dispersed on the surface of the Ti-Ag-Cu alloy. The micro-area potential differences between α-Ti and Ti_2_Ag might also affect the antibiofilm activity. In addition, the zeta potential [42] and wettability [43] affect bacterial adhesion. Through the limited experiments in this study, we revealed that the Ti-Ag-Cu alloys have the ability to suppress biofilm formation. Elucidation of this mechanism will be the subject of future studies.

### 4.4. Dental Application of the Ti-Ag-Cu Alloy

We succeeded in developing Ti-Ag-Cu alloys with excellent mechanical properties and antibiofilm activity, which was the purpose of this study. The alloy compositions used were 20Ag1Cu and 20Ag2Cu. These alloys satisfied the criteria for type 4 metallic materials. Superstructures and bridges made of these alloys are expected to prevent secondary caries and periodontitis owing to their antibiofilm activity. Furthermore, 20Ag1Cu and 20Ag2Cu can also be used for narrow-diameter implants, as their strength is comparable with those of pure Grade 4 Ti (550-750 MPa) [44] and ASTM/ASME Grade 4 Ti (550 MPa or higher) [45,46], which are materials used in the fabrication of narrow implants. Implants made from the Ti-Ag-Cu alloys will prevent peri-implantitis, owing to their antibiofilm activity. Copper cytotoxicity may be a concern for dental applications of Ti-Ag-Cu alloys; however, many studies conducted on binary Ti-Cu alloys for implant applications have shown good results [47,48,49]. Thus, Ti-Ag-Cu alloys may be suitable for use in the fabrication of dental applications, although the biocompatibility of the alloys should be investigated in the near future.

## 5. Conclusions

The strengths of 20Ag1Cu and 20Ag2Cu were significantly higher than that of Ti-20%Ag. The solid solution strengthening of Cu in α-Ti mainly contributed to the improvement in strength. These alloys showed excellent mechanical properties that are applicable to dental prostheses requiring high strength, such as long-span bridges and narrow-diameter implants. Furthermore, this alloy had an antibiofilm activity similar to that of the Ti-Ag alloy. Thus, the Ti-Ag-Cu alloys developed in this study are expected to be useful in preventing post-treatment oral diseases.

## Data Availability

The authors confirm that data supporting the findings of this study are available within the article.
